# Gastrointestinal Dysfunction in Critically Ill Patients With Traumatic Brain Injury: Clinical Implications and Putative Mechanisms: a Narrative Review

**DOI:** 10.1007/s11910-026-01500-7

**Published:** 2026-07-04

**Authors:** Ori J. Lieberman, Luisa Rojas-Valencia, Edilberto Amorim, Adam R. Ferguson, H. E. Hinson

**Affiliations:** 1 Department of Neurology, Weill Institute for Neurosciences, University of California San Francisco (UCSF), San Francisco, CA USA; 2 Brain and Spinal Injury Center (BASIC), Weill Institute for Neurosciences, San Francisco, CA USA; 3 Department of Neurological Surgery, Weill Institute for Neurosciences, University of California San Francisco (UCSF), San Francisco, CA USA; 4 San Francisco Veterans Affairs Healthcare System, San Francisco, CA USA

**Keywords:** Traumatic brain injury, Gut microbiome, Ileus, Nutrition, Neuroinflammation

## Abstract

**Purpose of the Review:**

Moderate to severe traumatic brain injury (TBI) requiring intensive care is associated with high morbidity, mortality and long-term disability. In addition to neurologic sequelae, TBI causes a systemic disease with associated injury to other organ systems, including the gastrointestinal (GI) tract. Here, we review the evidence that GI tract dysfunction occurs after TBI and discuss the clinical implications of GI tract dysfunction on the clinical care of TBI patients, including inadequate nutritional support, elevated risk of pneumonia, and a hyperactive inflammatory response.

**Recent Findings:**

We highlight recent findings that highlight putative mechanisms through which GI tract pathology may arise after TBI, including vagal nerve and enteric nervous system dysfunction, gut microbiome dysbiosis, sympathetic hyperactivity and iatrogenic injury. Finally, we highlight future approaches to target the GI tract that could improve outcomes in this critically ill patient population.

**Summary:**

In summary, we review the evidence supporting a role for GI tract dysfunction in the pathophysiology of critically ill TBI patients and highlight potential mechanisms through which GI tract dysfunction may worsen outcomes in this population.

## Introduction

Traumatic brain injury (TBI) represents a common and highly morbid disease [1]. Moderate to severe TBI requiring intensive care unit admission for close neurologic monitoring, mechanical ventilation, or administration of vasoactive medications comprises approximately 20% of all head injuries and can lead to long-term neurologic and physical disability, impaired level of arousal, and death [2–4].

Interestingly, head injury, even in the absence of polytrauma, can produce a systemic disease with impairment in multiple organ systems. Death and disability from non-neurologic organ dysfunction after TBI is common acutely [5] and in the months after injury [6]. A better understanding of the pathophysiology that drives these extracranial manifestations of head injury and identification of potential therapeutic strategies that mitigate these manifestations represents a potential avenue to improve outcomes in this vulnerable population.

While often overlooked, the gastrointestinal tract is impacted in 50% of patients with moderate or severe TBI [5–10]. Here, we present a narrative review of the the evidence for dysfunction of the GI tract after TBI, the clinical implications of GI tract dysregulation on TBI outcomes and potential mechanisms through which head injury may impact GI function. Although we will focus on clinical studies that describe these phenomena in the acute setting for patients with moderate to severe head injury, where applicable, preclinical studies that provide mechanistic insight will be highlighted. Finally, we detail future directions using novel approaches that exploit the crosstalk between the GI tract and central nervous system that have the potential to improve outcomes after TBI.

## Evidence for GI Tract Dysfunction after TBI

The earliest evidence of GI tract dysfunction after TBI came from reports of feeding intolerance. This was defined as elevated gastric residual volumes, aspiration, abdominal distension or diarrhea after initiation of enteral nutrition after TBI. Norton and colleagues reported a series of 23 patients with head injury and Glasgow Coma Scale (GCS) between 4 and 10 [7]. In this series, only seven patients tolerated enteral nutrition within 7 days of injury. Intolerance was associated with initial GCS and peak intracranial pressure. Similar findings have been reported from other centers and with modern enteral nutrition formulae [8–10].

Potential mechanisms for feeding intolerance include dysfunction of both the upper GI tract and small intestine. Oropharyngeal dysphagia is common acutely after head injury [11]. In addition, abnormal lower esophageal sphincter function has been reported within 72 h of moderate to severe TBI [12]. Delayed gastric emptying has been shown repeatedly in the acute period after TBI [13–16]. This is associated with delays in tolerance of enteral nutrition and is more severe in patients with higher intracranial pressure. Animal models of TBI demonstrate disparate evidence related to gastric emptying, with some showing delays [17] and others showing an association between elevated ICP and accelerated gastric emptying [18]. Data supporting abnormal function of the small intestine after TBI is predominantly derived from animal models. Early disruption of the intestinal mucosa and increased intestinal permeability has been shown as early as three hours after weight drop TBI in rats [19] and this persists for several days after injury [20, 21]. This does not seem to be dependent on intracranial lesion location. These permeability changes in the small intestine seem to occur across all segments and involve increased paracellular transport and a defect in tight junction integrity [19–21]In addition, small intestine contractility is delayed, an effect thought to be related to intestinal smooth muscle dysfunction [22] .Together, these data highlight a variety of potential anatomic and mechanistic explanations for intolerance of enteral nutrition after TBI.

## Clinical Implications of GI Dysfunction after TBI

**Fig. 1:**
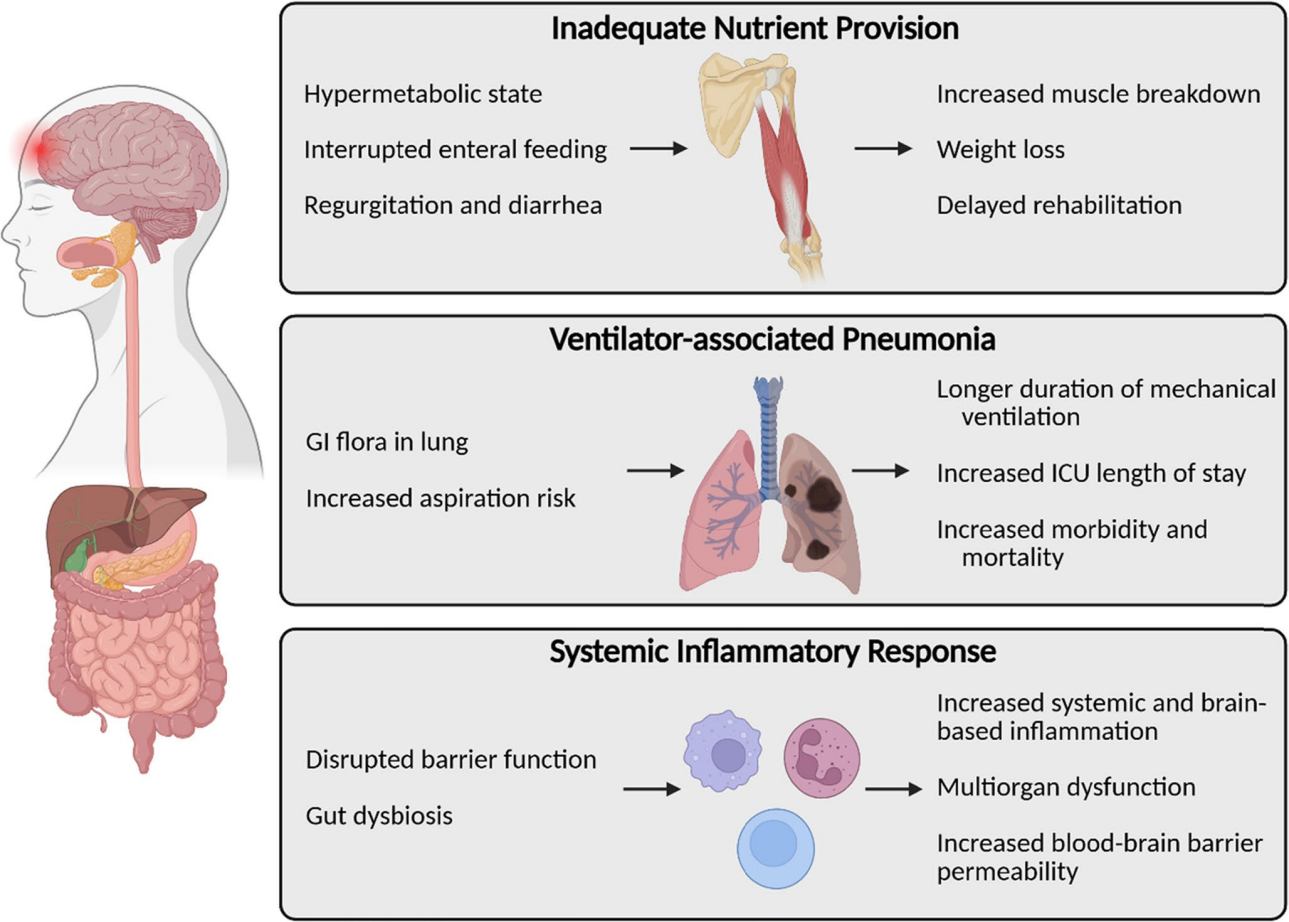
Consequences of GI tract dysfunction on TBI patients. Created in BioRender. Lieberman, O. (2026) https://BioRender.com/erxih05

There are several clinical consequences of GI tract dysfunction after TBI (Fig. 1). These include inadequate nutrient provision, aspiration and pulmonary infection, and an aggravated systemic inflammatory response. Together these complications affect many TBI patients during the first weeks after injury and exacerbate morbidity and mortality. In this section, we will review these and emphasize the role of GI dysfunction in causing preventable complications after TBI.

### Nutrient Provision

After moderate or severe TBI, many patients require assisted feeding for several weeks due to depressed level of arousal, oropharyngeal dysphagia or as a consequence of mechanical ventilation via endotracheal or tracheostomy tube [23]. Patients may be fed enterally via nasogastric, nasoduodenal, or gastrostomy feeding tubes or fed parenterally using Total Parenteral Nutrition (TPN).

The Brain Trauma Foundation guidelines [24, 25] provide guidance regarding calorie and protein provision, timing of initiation of nutrient provision and route of feeding. In large retrospective studies, about half of patients are not provided with recommended caloric or protein provisions during the first week after injury [23, 26, 27]. This is associated with delayed ICU and hospital discharge, increased risk of mortality, and worse functional outcomes 3–6 months after injury. These studies suggest that complications of enteral feeding, including regurgitation and aspiration, abdominal distention and diarrhea, are key factors in limiting nutrition delivery to patients with TBI during the acute period. Additional small randomized controlled trials have studied different approaches to bypass GI dysfunction-associated complications of enteral nutrition to promote early adequate nutrition delivery. These studies have highlighted a role for parenteral or modified enteral strategies, including the use of prokinetic agents such as metoclopramide or erythromycin, elemental nutrition formulae, and transpyloric feeding tubes, which may improve outcomes in TBI patients [28–34].

Inadequate nutrient provision poses a particular risk to patients who have suffered TBI because of the post-injury hypermetabolic state (reviewed in [35]). Recognized as early as the 1970’s [36], the hypermetabolic state after TBI can last for several weeks after injury and is associated with an enhanced basal metabolic rate (~ 150% of predicted) and negative nitrogen balance [35, 37, 38]. This catabolic state leads to weight loss during the hospital stay which can impact rehabilitation potential [35, 37, 39, 40]. Furthermore, patients with larger negative energy and nitrogen balances or delayed exposure to enteral nutrition have higher risk of in-hospital mortality, prolonged ICU and hospital lengths of stay and longer duration of mechanical ventilation [34, 35, 41–43].

Together, this suggests that impaired GI tract function after TBI limits the delivery of adequate nutrition and contributes to worse outcomes.

### Aspiration and Ventilator-associated Pneumonia

Pneumonia affects between 20 and 40% of severe TBI patients during the first month after injury [44, 45]. This can be associated with aspiration at the time of injury or ventilator-associated if it develops in a delayed fashion. The high incidence of pneumonia after TBI has been associated with a prolonged duration of mechanical ventilation as well as ICU and hospital length of stay [44–46]. Single center studies have suggested acute lung injury after TBI, of which a substantial fraction is due to nosocomial infection, is associated with higher mortality rates and worsened neurologic outcomes [45, 47, 48]. A particular challenge in this field is the lack of rigorous diagnostic criteria for ventilator-associated pneumonia, which leads to variable estimates of pneumonia incidence and difficulty defining attributable consequences [45, 49–51]. Importantly, reducing pneumonia incidence in TBI patients either with antibiotic prophylaxis or selective digestive decontamination improves functional outcomes and reduces mortality of acute brain injury, suggesting that this represents a potential target to mitigate secondary neurologic injury [52–54] .

Interestingly, rates of pneumonia are higher after TBI than other critical illnesses [49, 55, 56]. In addition to the depressed level of arousal, absent cough and gag reflex, and abnormal immune response after TBI, GI tract dysfunction has been proposed as a potential mechanism for the higher incidence of pneumonia in this population [44, 45, 57]. In critically ill patients without head injury, gastric feeding and diminished gastric emptying are associated with higher incidence of pneumonia and this may apply in TBI patients though it has not been demonstrated [51, 58, 59]. In further support of the relationship between GI tract dysfunction and ventilator-associated pneumonia, the microbial ecology of lower respiratory tract infection in the TBI population is dominated by GI flora [60, 61]. Together, this suggests that GI tract dysfunction may be linked to the high incidence of pneumonia in TBI patients.

Efforts to optimize enteral nutrition delivery have suggested that the contribution of GI tract dysfunction to pneumonia risk in TBI patients is modifiable. A randomized controlled trial of pre- versus post-pyloric enteric feeding in patients with TBI showed a reduction in pneumonia incidence [33]. Pro-kinetic agents, such as metoclopramide, have not been shown to reduce incidence of pneumonia in TBI patients [32], though metoclopramide does not seem to have a consistent effect on gastric motility in patients with TBI as it does in others with critical illness [31, 32]. Despite this available evidence, the Brain Trauma Foundation guidelines do not provide a strong recommendation for post-pyloric feeding to minimize pneumonia risk in TBI patients but highlight the need for additional studies [25]. In summary, reducing gastrointestinal tract dysfunction after TBI may minimize secondary neurologic injury and medical complications associated with pneumonia and improve outcomes in this population.

## Inflammatory Response after TBI

After TBI, a massive systemic and neuro-inflammatory response occurs [62, 63]. Centrally, the inflammatory response may be protective [64, 65]. However, the hyperactive systemic inflammatory response characterized by increased blood levels of several cytokines, including TNFα, IL-1β, IL-6, IL-8 and IL-10, is more pronounced in patients who die or have poor neurologic outcome [66–72]. The association between an exaggerated systemic inflammatory response and worse outcome after TBI is thought to be driven not only by secondary neurologic injury, but also by multiorgan dysfunction [5, 72–75].

The GI tract has been proposed as a driver of multiorgan dysfunction in critical illness generally via stimulation of a similar hyperinflammatory response [76, 77]. Splanchnic vasoconstriction in the setting of sepsis or polytrauma is thought to lead to gut ischemia-reperfusion injury, a local inflammatory response in the small intestine, and increase gut epithelial permeability [78, 79]. This may further stimulate a pro-inflammatory response via translocation of gut microbes or endotoxin into the portal and systemic circulation, though this has yet to be shown in humans [19].

In animal models after TBI, significant functional and histopathological changes have been described in the small intestine leading to a local pro-inflammatory response and increased gut permeability. In a series of studies in the mid-2000’s, Hang et al. demonstrated morphologic changes to villi of the small intestine within 3 days of TBI, enhanced NF-κB activation and cytokine production, and increased intestinal permeability due to disruption of tight junctions [19, 80, 81]. This finding has been corroborated in fly models of TBI [82]. Interestingly, reversal of the increase in intestinal permeability after TBI reduced circulating levels of TNFα in mice [83–86].

The gut microbiome is essential for the proper development of the immune system and ensuring an appropriate inflammatory response in both health and disease [87, 88]. In addition to the alterations in intestinal permeability and the local intestinal inflammatory milieu described above, changes in the composition of the gut microbiome after TBI have been described in animal models [89–95]. Although there is variability in the timing after injury and specific changes in gut microbiome composition described in each of these studies, a consensus has emerged that TBI leads to decreases gut microbial diversity and the proliferation of potentially pathogenic bacterial species [96, 97]. Notably, measurement of gut microbiome composition has focused on stool, likely reflecting the community present in the cecum and not in the small intestine, where most pathological changes have been noted to occur [19]. TBI-associated changes in the gut microbiota have been causally linked with increased lesion volume, perilesional inflammatory changes and more severe behavioral deficits [96, 98–103]. Two distinct mechanisms have been proposed to mediate the effect of gut microbiome on TBI severity and associated neuroinflammation: (1) altered production of microbial metabolites, such as short-chain fatty acids that act distally to modulate the brain endothelium and microglia [92], or (2) modulation of local intestinal inflammatory responses that then traffic to the brain [103, 104]. These studies suggest that the inflammatory response after TBI in animal models may be modulated by the gut microbiome, though confirmatory studies in humans have not yet been performed.

Evidence of increased intestinal permeability and gut dysbiosis has been suggested in humans after TBI, though large-scale studies in isolated head injury have not been conducted as they have in polytrauma or sepsis [78, 105–107]. Together, this data highlights the possibility that the gut may act as a driver for the systemic inflammatory response after TBI, though additional studies are needed to better define these mechanisms in humans.

## Potential Mechanisms for GI Dysfunction after TBI

**Fig. 2:**
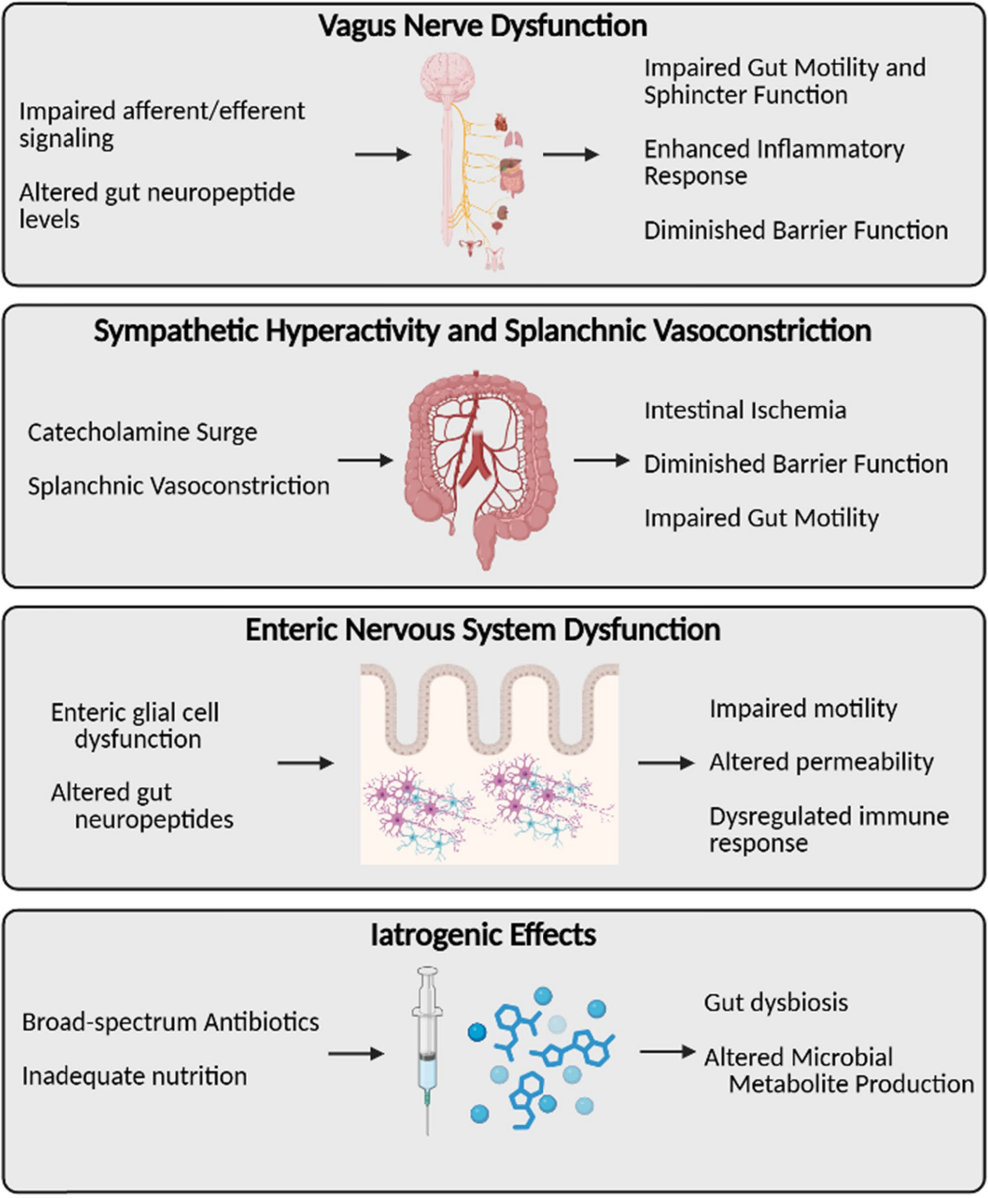
Putative mechanisms that drive GI tract dysfunction after TBI. Created in BioRender. Lieberman, O. (2026) https://BioRender.com/b1pfegr

The high prevalence of GI tract dysfunction after head injury and its broad clinical implications raise the question of what the underlying mechanisms could be. Several putative mechanisms have been proposed and we will review each here (Fig. 2). Notably, each of these are interconnected and may impact GI tract function after TBI in an overlapping manner.

### Vagus Nerve

Gastrointestinal motility, sphincter function, intestinal epithelial cell barrier function and nutrient absorption are controlled in part by the parasympathetic nervous system via the vagus nerve [108]. In the healthy individual, vagal afferents innervating the GI tract sense both chemical and mechanical stimuli from the stomach, small intestine and colon through the splenic flexure and relay this to the nucleus tractus solitarius (NTS), where interneurons modulate the dorsal motor nucleus of the vagal nerve (DMV) and vagal efferents that provide top-down control gastrointestinal tract function [109]. In addition to GI motility, the vagus nerve dampens the local inflammatory milieu in the gut via cholinergic signaling [110–112]. This suggests that the vagal nerve, both via its afferent and efferent limbs, could serve as a key driver of GI tract dysfunction after TBI.

In animal models, both the afferent and efferent limbs of the vagal nerve are impacted after TBI. Neuropeptides released by enteroendocrine cells, such as cholecystokinin (CCK), vasoactive intestinal peptide (VIP), ghrelin, and glucagon-like peptide (GLP), in the gut are sensed by vagal afferents [113]. Both jejunal and plasma levels of CCK, VIP and GLP after experimental TBI are altered [83, 86, 102, 114], suggesting impaired enteroendocrine input onto vagal afferents may contribute to GI dysfunction in this setting. In addition, vagal afferents can directly sense gut microbe metabolites [115], which may be diminished after TBI [104]. Vagal efferent activity may also be disrupted after TBI}. TNFα, a pro-inflammatory cytokine whose systemic and central production surges after TBI [67, 70], can modulate synaptic plasticity and neurotransmission in the NTS and DMV, leading to abnormal vagal efferent activity [116–118].

In addition to these studies in animal models, abnormal activity of the vagus nerve can be inferred in humans by measurement of the variability in heart rate (HRV), which is under prominent parasympathetic control [119–121]. After TBI, patients with HRV parameters that suggest elevated parasympathetic tone are less likely to have a favorable outcome [122, 123], but no study linking HRV to GI dysfunction has been performed in this population.

Modulation of vagal nerve activity via invasive or non-invasive stimulation may improve functional outcomes after TBI [124]. This may occur via modulation of cerebral edema, blood-brain barrier permeability and the inflammatory response [125–128]. Interestingly, Bansal et al. has shown that vagal nerve stimulation reduces the intestinal and systemic inflammatory response to TBI and this is required for improvement in behavioral assays after TBI in rodents [84, 86].

These data suggest that impaired vagal nerve activity may be a key mechanism that drives GI tract dysfunction after TBI and may provide a therapeutic target to improve outcomes in this population.

### Sympathetic Hyperactivity and Splanchnic Vasoconstriction

In sepsis and after trauma, a catecholamine surge drives vasoconstriction of splanchnic vasculature that supplies the stomach and small intestine [129–131]. This allows for the redistribution of blood supply to other organs that are critical for survival in a stressed state, such as the brain, kidneys and heart [77]. The relative ischemia and subsequent reperfusion injury leads to intestinal pathology including villus blunting, enhanced permeability and impaired motility [132–134]. In addition, changes in the composition of the gut microbiome have been linked to intestinal ischemia/reperfusion injury in other settings [135] .

Catecholamine surges have been observed after TBI [136] and are associated with worse outcome [137]. No study has linked circulating catecholamine levels or other measures of sympathetic nervous system activity with GI tract dysfunction after TBI. However, the degree of inflammatory response after TBI is correlated with circulating catecholamine levels [138]. Similarly, splanchnic perfusion after TBI has not yet been assessed. Blockade of catecholaminergic signaling with beta blockers such as propranolol after TBI has been evaluated for prevention of paroxysmal sympathetic hyperactivity [139, 140]. Whether this impacts GI tract pathology remains unknown. Future studies should investigate the relationship between catecholamine release and splanchnic vasoconstriction with GI pathology after TBI.

### Enteric Nervous System (ENS)

The enteric nervous system (ENS) is a collection of neurons and glial cells that reside within the myenteric and submucosal plexi, which are present throughout the GI tract [141]. Neurons in these plexi not only control gastrointestinal contractility via connection with varying muscle groups but also contribute to regulation of intestinal immune cells [142]. They are until the control of local neuropeptides, such as CCK, released by enteroendocrine cells and top-down control from the parasympathetic and sympathetic nervous systems. In other disease states, dysfunction of the enteric nervous system leads to abnormal motility, inflammatory responses, permeability and nutrient absorption (reviewed in [143]). In animal models of TBI, activation of enteric glial cells (EGCs) in the colon is acutely decreased in the first day, but then rebounds at 28 days [96]. Similarly, EGCs modulate the response to toxic and ischemic/reperfusion injury in the intestine [144]. Although the exact role of the ENS in TBI-related GI dysfunction remains unclear, these studies suggest the potential for pathological ENS activity to mediate some of the effect of TBI on the GI tract.

### Iatrogenic Injury – Enteral Nutrition and Antibiotics

Iatrogenic injury after TBI may lead to GI tract dysfunction. Delayed administration of enteral nutrition is common in severe TBI [23] and is associated with loss of intestinal barrier function [145–148]. The composition of the gut microbiome also depends on nutritional intake [149, 150]. In addition, administration of broad-spectrum antibiotics during critical illness can impact the gut microbiota [107, 151, 152], leading to GI tract pathology. Antibiotic use is common in critically ill patients with TBI [153, 154] and may contribute to alterations in the gut microbiome after TBI. Finally, common ICU interventions, including exposure to sedation and opiates, can impact GI tract function, but their impact in TBI patients specifically has not been studied. Future studies that define the impact of enteral nutrition and antibiotic exposure on GI tract function after TBI may allow for a more comprehensive understanding of the impact of these interventions on patients with TBI.

## Limitations of the Evidence Base Describing GI Dysfunction after TBI

Several methodological and conceptual limitations are present in the literature describing gastrointestinal dysfunction after TBI and its consequences. First, many descriptive clinical studies of feeding intolerance, metabolic need, ileus and gastric emptying have not been performed in modern cohorts treated according to the most updated clinical guidelines. Second, clinical studies of GI dysfunction in TBI patients at times include heterogeneous patient populations with a variety of injury severities, surgical interventions, and degree of polytrauma. Third, animal models of TBI used for mechanistic studies of GI dysfunction do not fully replicate the critical illness associated with severe TBI in humans. Finally, the causal association between GI dysfunction and outcome after TBI remains to be fully assessed in clinical trials.

## Conclusions

Taken together, this literature provides strong support for the notion that disruption of normal GI tract physiology after TBI represents a tantalizing opportunity to intervene and improve outcomes in this vulnerable population. We suggest three research priorities going forward that could allow translation of these findings into clinical care: (A) generation of additional evidence to optimize the delivery of enteral nutrition shortly after injury, (B) a more detailed characterization of the gut microbiome and its metabolites after head injury that test hypotheses from animal models regarding its role in TBI pathogenesis, and (C) additional trials aimed at testing interventions aimed at minimizing GI tract dysfunction and the inflammatory response after TBI. Advances in each of these domains have the potential to change the acute care of the patient with TBI and leverage intervention on extracranial organs to minimize the neurologic and functional consequences of head injury.

## Key References

- Norton JA, Ott LG, McClain C, Adams L, Dempsey RJ, Haack D, et al. Intolerance to enteral feeding in the brain-injured patient. J Neurosurg. 1988;68:62–6.
  - ○ This is one of the original descriptions of feeding intolerance in TBI patients.
- Krakau K, Omne-Pontén M, Karlsson T, Borg J. Metabolism and nutrition in patients with moderate and severe traumatic brain injury: A systematic review. Brain Inj. 2006;20:345–67.
  - ○ This is a definitive review that highlights the changes in metabolism after TBI.
- Prieto-Alvarado DE, Parada-Gereda HM, Molano D, Martinez YL, Tafurt GPR, Masclans J-R. Risk factors and outcomes of ventilator-associated pneumonia in patients with traumatic brain injury: A systematic review and meta-analysis. J Crit Care. 2025;85:154922.
  - ○ This review highlights GI tract dysfunction as a risk factor for pneumonia after TBI.
- Hang C-H, Shi J-X, Li J-S, Wu W, Yin H-X. Alterations of intestinal mucosa structure and barrier function following traumatic brain injury in rats. World J Gastroenterol. 2003;9:2776–81.
  - ○ This manuscript highlights the effect of TBI on the intestinal mucosa and its structure in rodents.
- Bansal V, Costantini T, Ryu SY, Peterson C, Loomis W, Putnam J, et al. Stimulating the Central Nervous System to Prevent Intestinal Dysfunction After Traumatic Brain Injury. J Trauma: Inj, Infect, Crit Care. 2010;68:1059–64.
  - ○ This manuscript provides proof of principle in an animal model that GI dysfunction can be targeted after TBI to improve outcomes.

## Data Availability

No datasets were generated or analysed during the current study.
